# Supplementary material for: Biliary NIK promotes ductular reaction and liver injury and fibrosis in mice
Source: Nat Commun. 2022 Aug 30;13:5111. doi: 10.1038/s41467-022-32575-8 (PMC9427946; doi:10.1038/s41467-022-32575-8)
Supplement: Supplementary file 1 — Supplementary Information [file 41467_2022_32575_MOESM1_ESM.pdf]

# Supplementary Information

# Biliary NIK promotes ductular reaction and liver injury and fibrosis in mice

Zhiguo Zhang, Xiao Zhong, Hong Shen, Liang Sheng, Suthat Liangpunsakul,

Anna S. Lok, M Bishr Omary, Shaomeng Wang, Liangyou Rui

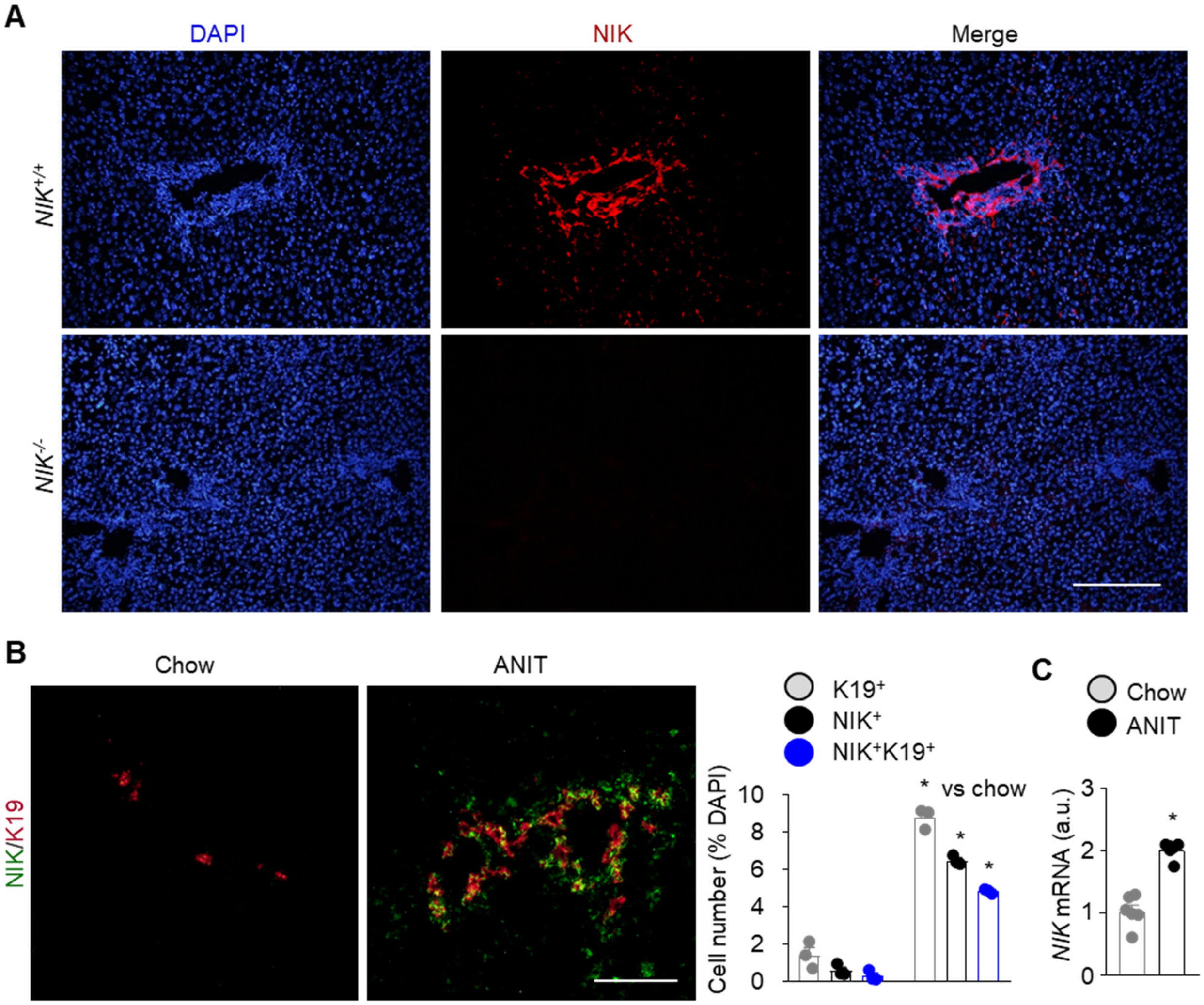


**Supplementary Figure 1. ANIT feeding upregulates biliary NIK in mice. (A)** *NIK*$^{+/+}$ and *NIK*$^{-/-}$ male mice were fed a DDC diet for 2 weeks. Liver sections were stained with anti-NIK antibody. Representative results were from 3 independently experiments. Scale bar: 200 µm. **(B-C)** C57BL/6J male mice were fed an ANIT diet for 3 weeks. **(B)** Liver sections were immunostained with antibodies to NIK and K19. NIK$^{+}$K19$^{+}$ cells were counted and normalized to total cells (n=3 mice per group). Scale bar: 200 µm. **(C)** Liver NIK expression was measured by qPCR (normalized to 36B4 levels, n=6 mice per group). Data are presented as mean ± SEM. *p<0.05, 2-tailed student's *t* test. Source data are provided as a Source Data file.

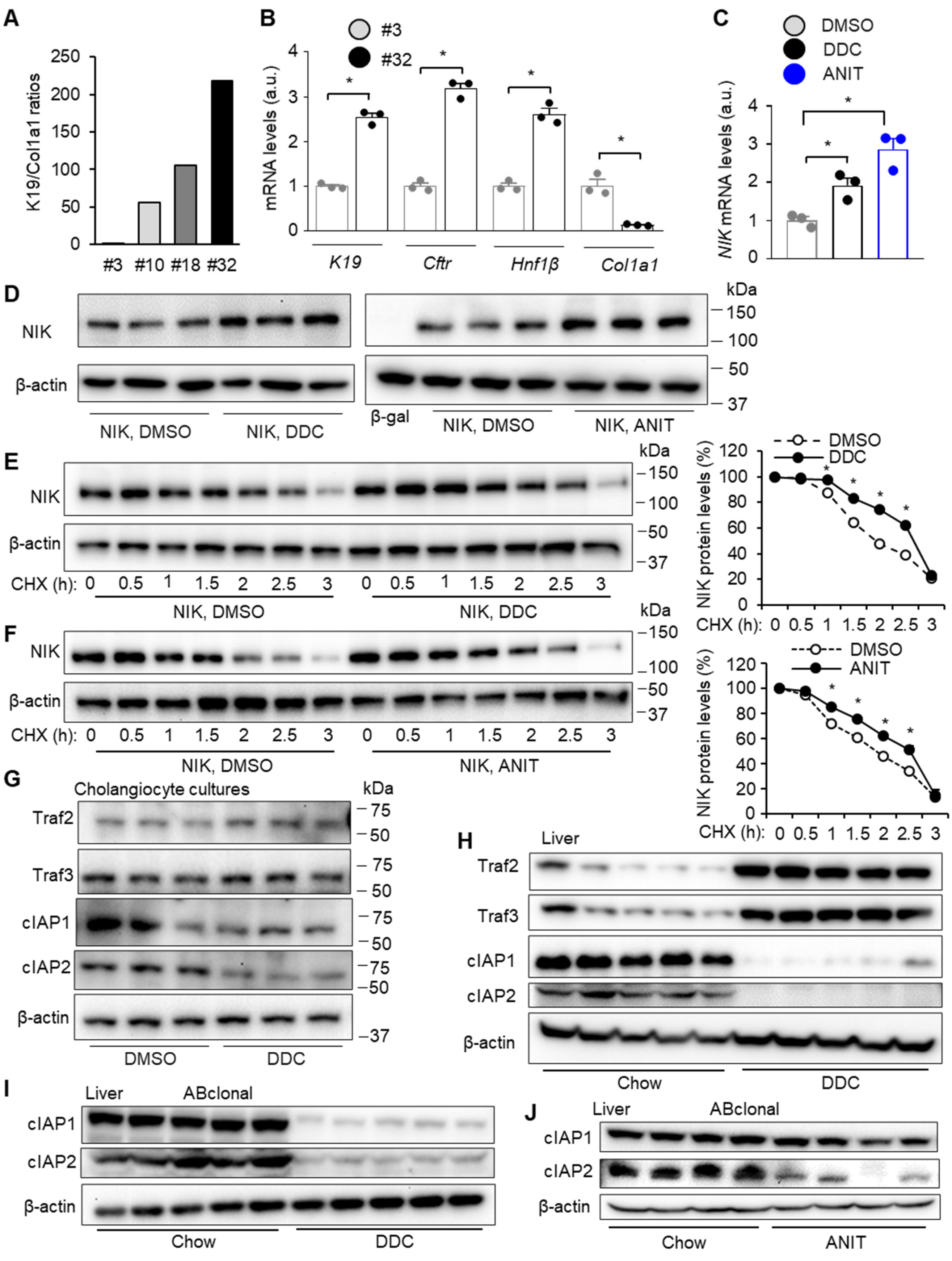
A
K19/Col1a1 ratios
250
200
150
100
50
0
#3
#10
#18
#32
B
#3
#32
mRNA levels (a.u.)
4
3
2
1
0
*
K19
Cftr
Hnf1β
Col1a1
C
DMSO
DDC
ANIT
NIK mRNA levels (a.u.)
4
3
2
1
0
*
D
NIK
β-actin
NIK, DMSO
NIK, DDC
kDa
150
100
50
37
β-gal
NIK, DMSO
NIK, ANIT
E
NIK
β-actin
kDa
150
100
50
37
CHX (h): 0 0.5 1 1.5 2 2.5 3 0 0.5 1 1.5 2 2.5 3
NIK, DMSO
NIK, DDC
DMSO
DDC
NIK protein levels (%)
120
100
80
60
40
20
0
CHX (h): 0 0.5 1 1.5 2 2.5 3
F
NIK
β-actin
kDa
150
100
50
37
CHX (h): 0 0.5 1 1.5 2 2.5 3 0 0.5 1 1.5 2 2.5 3
NIK, DMSO
NIK, ANIT
DMSO
ANIT
NIK protein levels (%)
120
100
80
60
40
20
0
CHX (h): 0 0.5 1 1.5 2 2.5 3
G
Cholangiocyte cultures
kDa
Traf2
Traf3
cIAP1
cIAP2
β-actin
75
50
37
DMSO
DDC
H
Liver
Traf2
Traf3
cIAP1
cIAP2
β-actin
Chow
DDC
I
Liver
ABclonal
cIAP1
cIAP2
β-actin
Chow
DDC
J
Liver
ABclonal
cIAP1
cIAP2
β-actin
Chow
ANIT

**Supplementary Figure 2. DDC and ANIT increase the expression and stability of cholangiocyte NIK. (A-B)** The biliary tree was isolated from *NIK*$^{f/f}$ male mice and dissociated into individual cells using collagenase. The cells were immortalized using E1A lentiviral vectors. Individual lines were isolated. **(A)** K19 and Col1a1 expression was measured by qPCR (normalized to 36B4) to calculate K19/Col1a1 ratios. **(B)** Gene expression in lines #3 and #32 (normalized to 36B4). N=3 repeats per group. **(C)** Cholangiocyte cultures (line #32) were stimulated with DDC (100 uM), ANIT (50 uM), or DMSO vehicle for 24 h. *NIK* mRNA levels were measured by qPCR and normalized to 36B4 (n=3 repeats per group). **(D)** Cholangiocyte cultures were transduced with β-gal or NIK adenoviral vectors and subsequently treated with DDC (100 uM), ANIT (50 uM) or DMSO vehicle for 24 h. Cell extracts were immunoblotted with antibodies to NIK and β-actin (each lane represents an independent repeat). **(E-F)** Cholangiocyte cultures were transduced with NIK adenoviral vectors, pretreated with DDC (100 uM), ANIT (50 uM), or DMSO for 4 h, and then treated with cycloheximide (100 ug/ml) for the indicated times. Cell extracts were immunoblotted with antibodies to NIK and β-actin. NIK levels were normalized to β-actin and presented as % of initial (n=3 repeats per point). **(G)** Cholangiocyte cultures were treated with DDC (100 uM) or DMSO for 24 h. Cell extracts were immunoblotted with the indicated antibodies (each lane represents an individual repeat). **(H-J)** *NIK*$^{f/f}$ male mice were fed a chow, DDC (4 weeks), or ANIT diet (3 weeks). Liver extracts were immunoblotted with the indicated antibodies (each lane represents an individual animal). Antibodies to cIAP1 and cIAP2 were from CST and Santa Cruz **(H)** and ABclonal **(I-J)**. Data are presented as mean ± SEM. *$p<0.05$, 2-tailed student's *t* test **(B)** and 1-way **(C)** or 2-way **(E-F)** ANOVA. Source data are provided as a Source Data file.

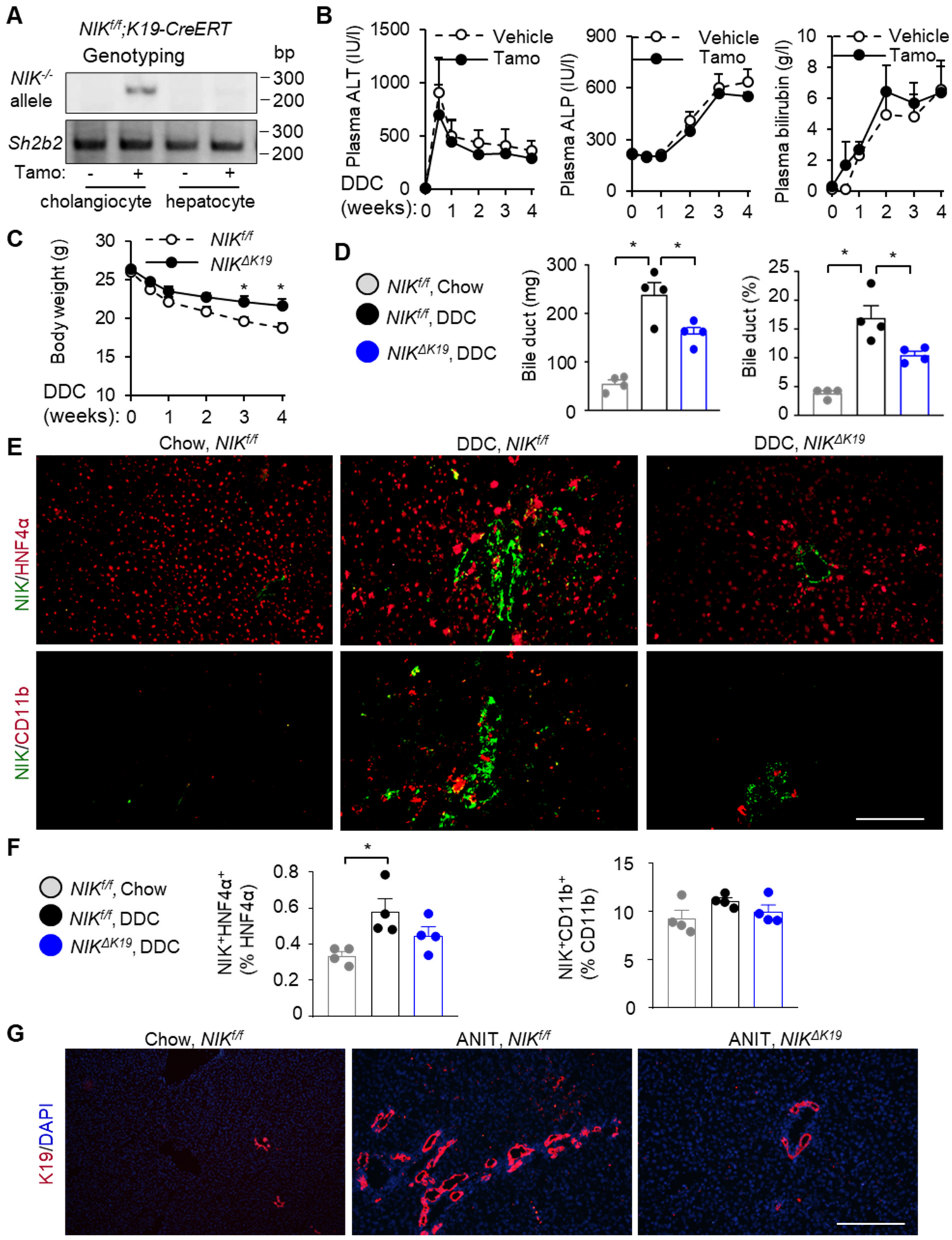


**Supplementary Figure 3. Ablation of biliary NIK protects against DDC- and ANIT-induced ductular reaction. (A)** Primary cholangiocyte and hepatocytes were prepared from *NIK*$^{f/f}$*;K19-*

*CreERT* mice and treated with or without tamoxifen for 24 h. Genomic DNA was isolated for PCR-based genotyping of *NIK* and *Sh2b2* (one repeat). The *NIK* primers amplify the disrupted, but not wild-type, *NIK* allele. **(B)** $NIK^{f/f}$ (not containing *K19-CreERT*) male mice (8 weeks) were treated with tamoxifen (Tamo) or oil vehicles and fed a DDC diet. Body weight and plasma ALT, ALP, and total bilirubin were measured (4 mice per group). **(C-F)** $NIK^{\Delta K19}$ and $NIK^{f/f}$ male mice were fed a DDC diet for 4 weeks. **(C)** Body weight: $NIK^{f/f}$: n=10 mice, $NIK^{\Delta K19}$: n=11 mice. **(D)** Intrahepatic biliary tree weight (normalized to liver weight, n=4 mice per group). **(E-F)** Liver sections were stained with the indicated antibodies. Individual subpopulations were counted (n=4 mice per group). Scale bar: 200 µm. **(G)** $NIK^{\Delta K19}$ and $NIK^{f/f}$ male mice were fed an ANIT or chow diet for 3 weeks. Liver sections were stained with anti-K19 antibody (repeated 3 times). Scale bar: 200 µm. Data are presented as mean ± SEM. *p<0.05, 2-way **(C)** and 1-way **(D, F)** ANOVA. Source data are provided as a Source Data file.

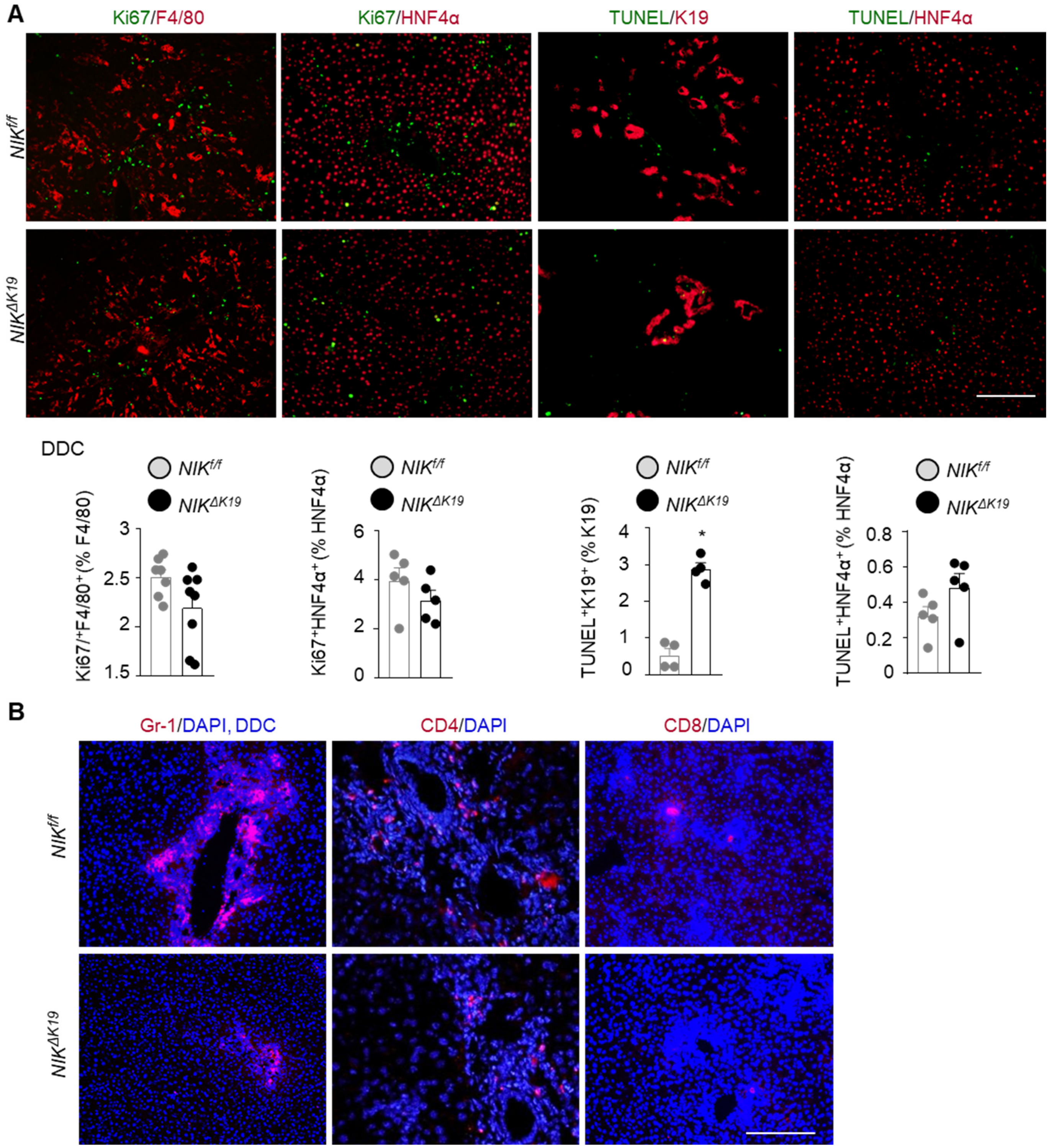


**Supplementary Figure 4. Ablation of biliary NIK influences liver cell proliferation and death.** *NIK*$^{\Delta K19}$ and *NIK*$^{f/f}$ male mice were fed a DDC diet for 4 weeks. Liver sections were stained with the indicated antibodies. Individual subpopulations were counted. Ki67$^{+}$F4/80$^{+}$: *NIK*$^{f/f}$: n=7 mice, *NIK*$^{\Delta K19}$: n=8 mice; Ki67$^{+}$HNF4α$^{+}$: n=5 mice per group; TUNEL$^{+}$K19$^{+}$: n=4 mice per group; TUNEL$^{+}$HNF4α$^{+}$:

n=5 mice per group. Scale bar: 200 μm. Data are presented as mean ± SEM. *p<0.05, 2-tailed student's *t* test. Source data are provided as a Source Data file.

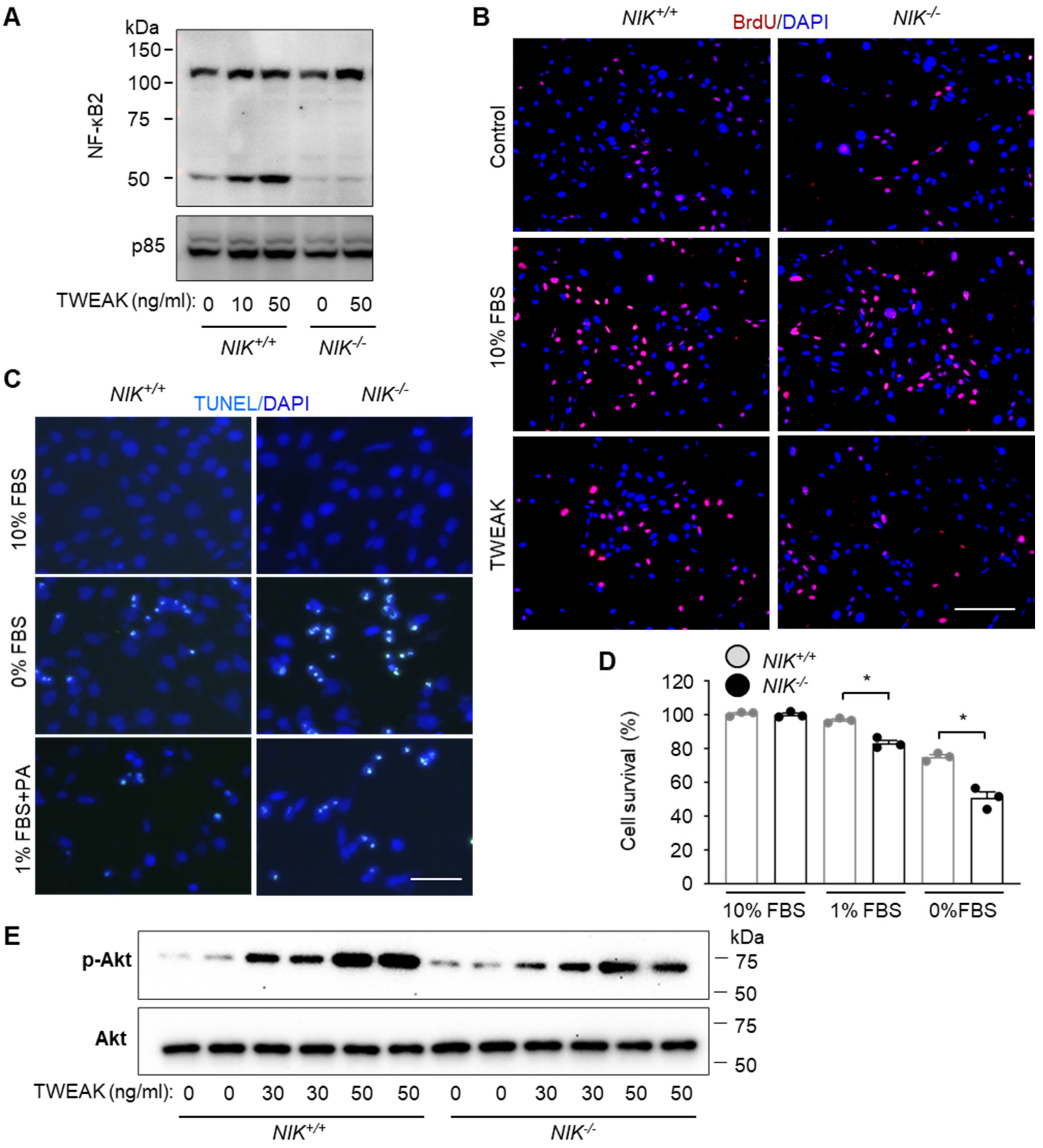


**Supplementary Figure 5. NIK directly regulates cholangiocyte proliferation and death. (A)** Cholangiocyte cultures were stimulated with TWEAK for 12 h. Cell extracts were immunoblotted with the indicated antibodies (one repeat). **(B)** Cholangiocyte cultures were deprived of serum overnight,

stimulated for 14 h with 10% FBS or TWEAK (10 ng/ml) in the presence of BrdU, and immunostained with anti-BrdU antibody (repeated >4 times). Scale bar: 200 μm. **(C)** Cholangiocyte cultures were deprived of serum or treated with 200 μM palmitate (PA) for 24 h and stained with TUNEL reagents. Scale bar: 200 μm. **(D)** $NIK^{+/+}$ and $NIK^{-/-}$ cholangiocytes were deprived of serum for 25 h. Cell viability was measured by MTT (n=3 repeats per group). **(E)** Cholangiocyte cultures were stimulated with TWEAK for 16 h. Cell extracts were immunoblotted with antibodies to phospho-Akt (pSer473) or Akt (repeated 3 times). Data are presented as mean ± SEM. *p<0.05, 2-tailed student's *t* test. Source data are provided as a Source Data file.

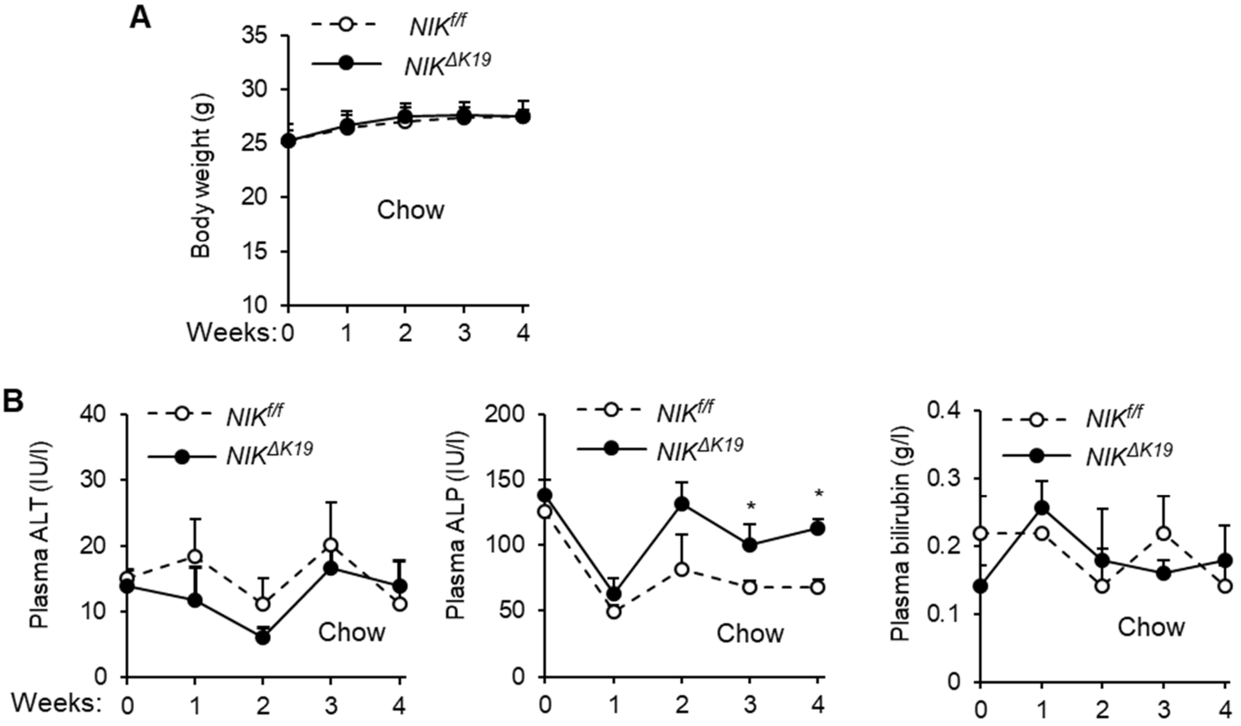


**Supplementary Figure 6. Characterization of $NIK^{\Delta K19}$ and $NIK^{f/f}$ males on chow. (A-B)** $NIK^{\Delta K19}$ and $NIK^{f/f}$ male mice were fed a chow diet. Body weight and plasma ALT, ALP and total bilirubin were measured (4 mice per group). Data are presented as mean ± SEM. *p<0.05, 2-way ANOVA. Source data are provided as a Source Data file.

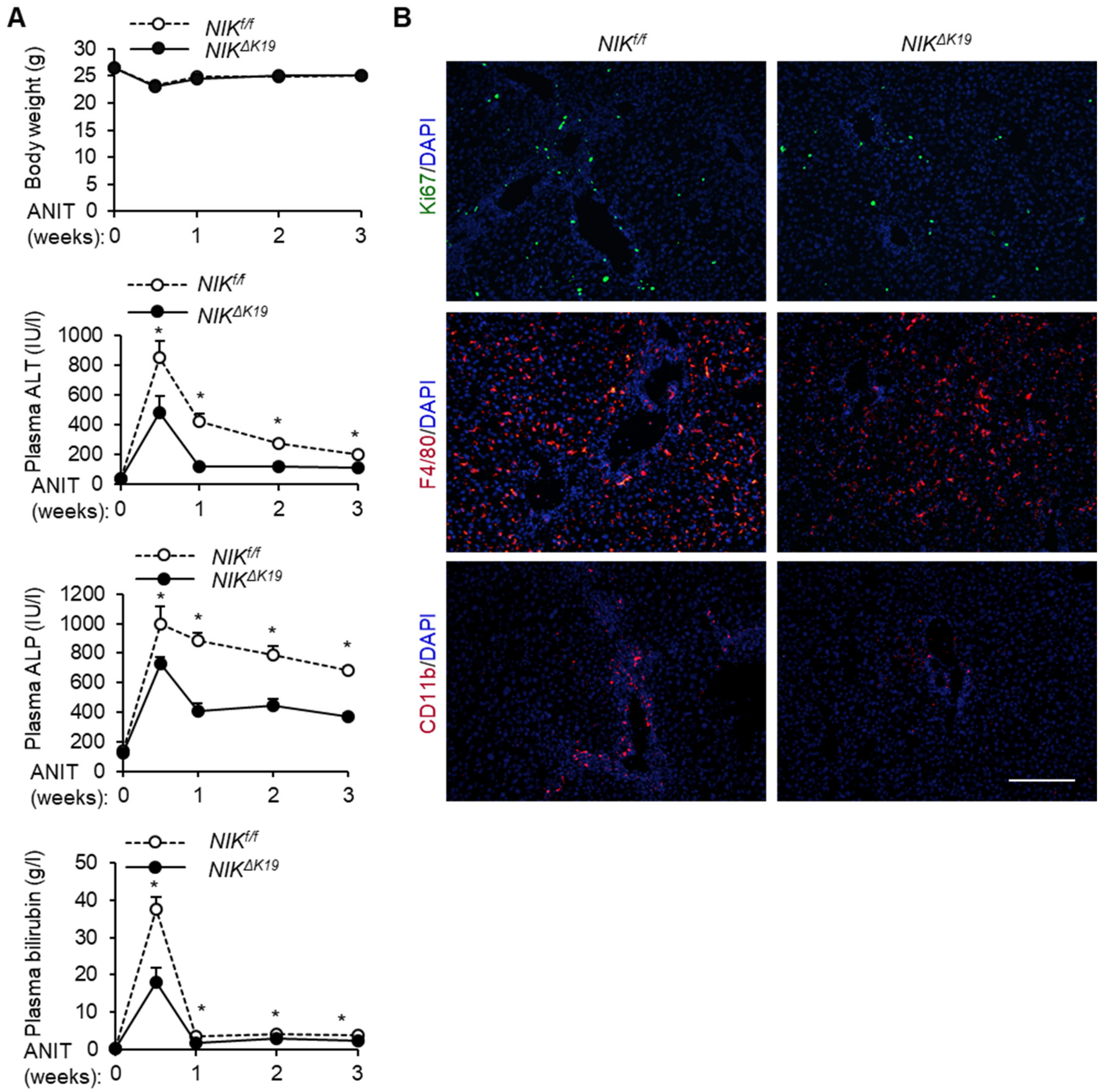


**Supplementary Figure 7. *NIK$^{ΔK19}$* mice are resistant to ANIT-induced cholestasis.** *NIK$^{ΔK19}$* (n=6 mice) and *NIK$^{f/f}$* (n=7 mice) males were fed an ANIT diet for 3 weeks. **(A)** Body weight and plasma ALT, ALP, and bilirubin levels. **(B)** Liver sections were stained with the indicated antibodies (repeated 4 times). Scale bar: 200 μm. Data are presented as mean ± SEM. *p<0.05, 2-way ANOVA. Source data are provided as a Source Data file.

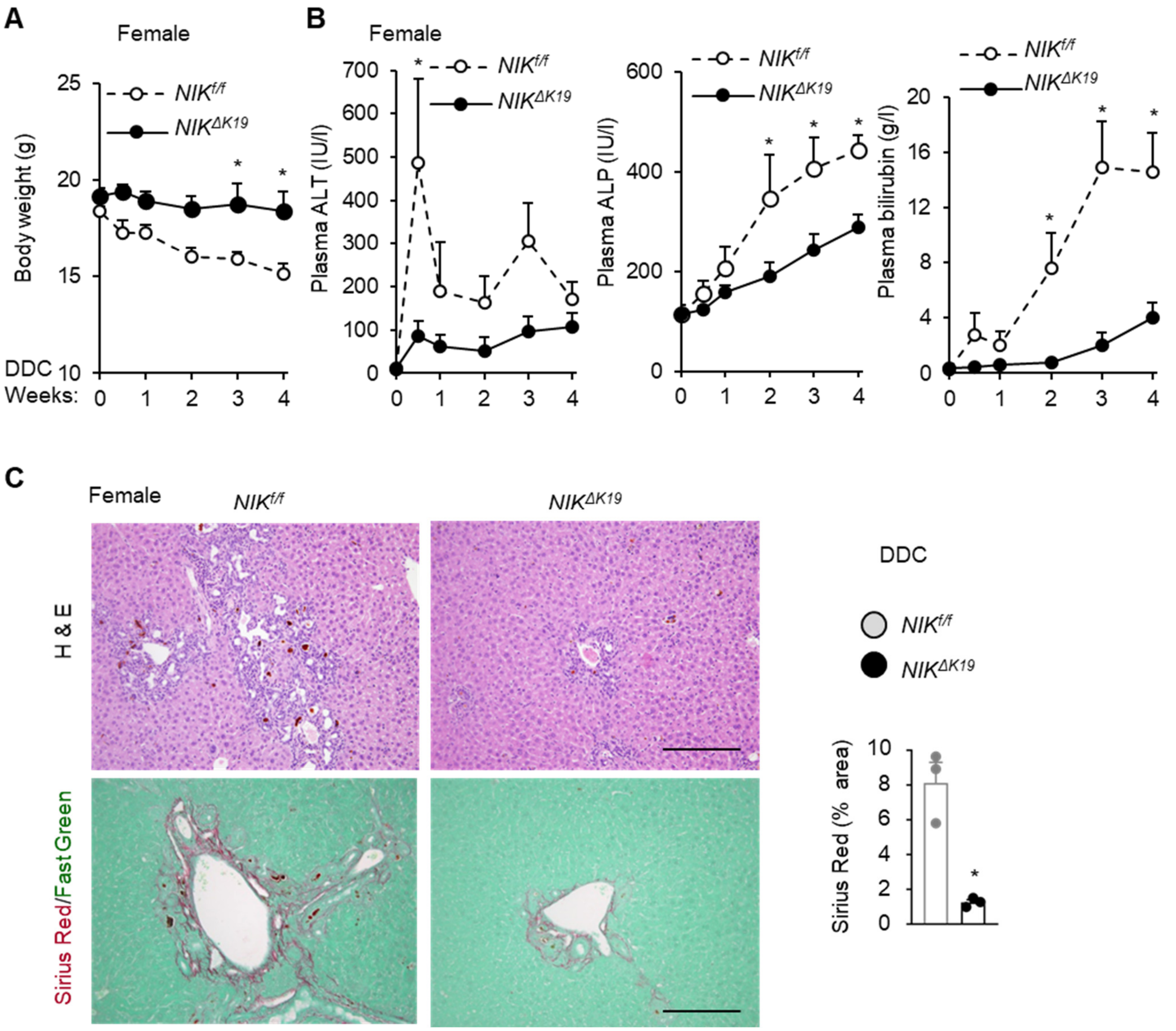


**Supplementary Figure 8. *NIK$^{\Delta K19}$* female mice are resistant to DDC-induced liver injury.** *NIK$^{\Delta K19}$* and *NIK$^{f/f}$* female mice were fed a DDC diet for 4 weeks. **(A-B)** Body weight and plasma ALT, ALP and total bilirubin levels. *NIK$^{\Delta K19}$*: n=5 mice, *NIK$^{f/f}$*: n=4 mice. (**C**) H&E and Sirius red staining of liver sections. Sirius red area was quantified and normalized to the total area (n=3 mice per group). Scale bar: 200 µm. Data are presented as mean ± SEM. *p<0.05, 2-way ANOVA (**A**, **B**) and 2-tailed student's *t* test (**C**). Source data are provided as a Source Data file.

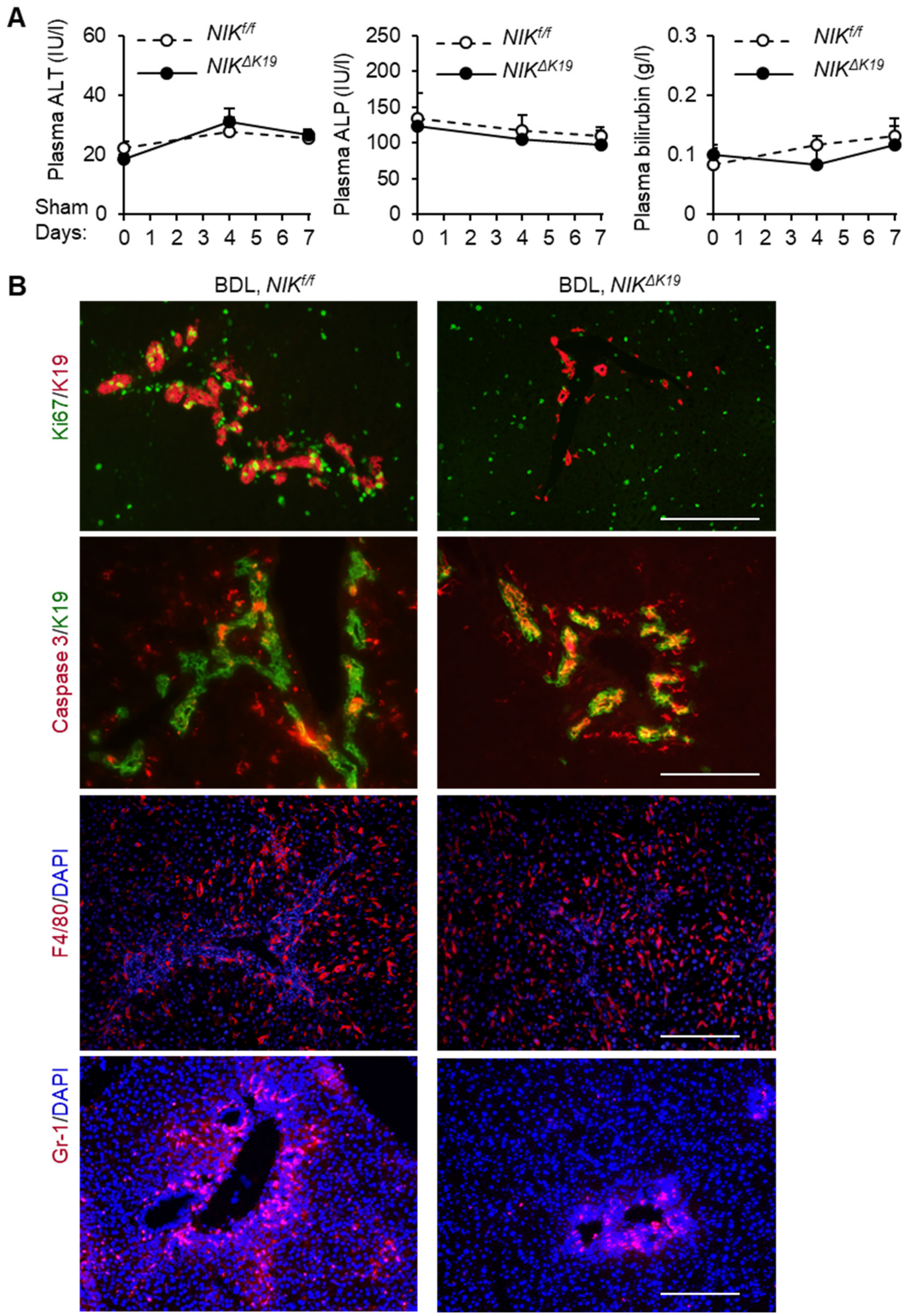


**Supplementary Figure 9. Ablation of biliary NIK attenuates BDL-induced cholangiocyte proliferation while increasing cholangiocyte apoptosis. (A)** Sham surgery was performed on *NIK*$^{ΔK19}$ and *NIK*$^{f/f}$ male mice (8 weeks). Plasma ALT, ALP and total bilirubin levels were measured (n=3 mice per group). (**B**) BDL was performed on *NIK*$^{ΔK19}$ and *NIK*$^{f/f}$ male mice (8 weeks). Liver sections were prepared 7 days post BDL and immunostained with the indicated antibodies (repeated

3-4 times). Scale bar: 200 µm. Data are presented as mean ± SEM. Source data are provided as a Source Data file.

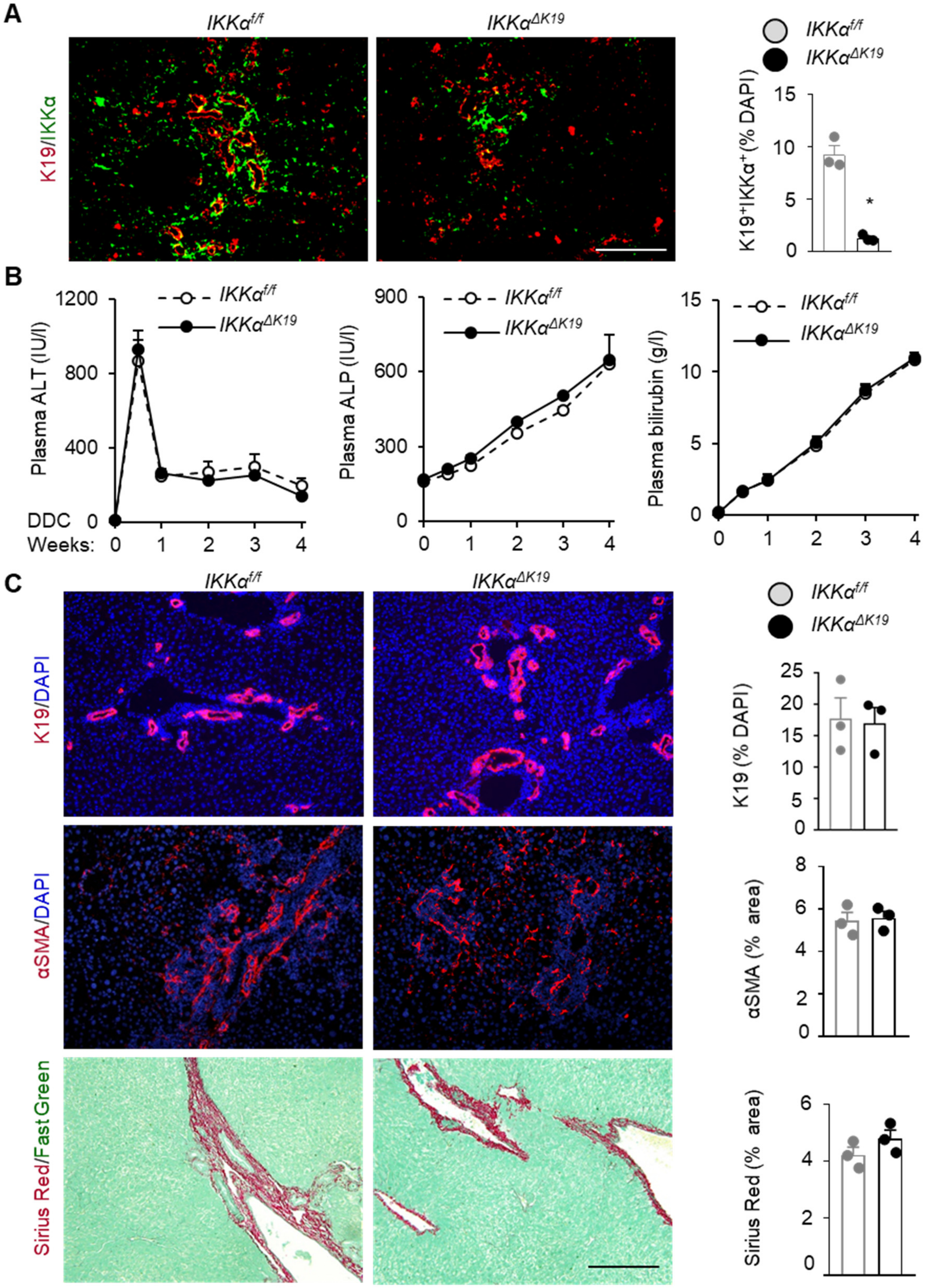

**Supplementary Figure 10. Deletion of biliary *IKKα* does not affect DDC-induced ductular reaction and liver injury.** *IKKα*$^{\Delta K19}$ (n=3 mice) and *IKKα*$^{f/f}$ (n=3 mice) males were fed a DDC diet for 4 weeks. **(A)** Liver sections were immunostained with antibodies to IKKα and K19. IKKα$^{+}$K19$^{+}$ cells were counted and normalized to total cells. Scale bar: 200 μm. **(B)** Plasma ALT, ALP, and total bilirubin levels. **(C)** Liver sections were stained with antibodies to K19 and αSMA or with Sirius red. K19$^{+}$ cells were normalized to total cells. αSMA$^{+}$ cells and Sirius red$^{+}$ area were normalized to total areas. Scale bar: 200 μm. Data are presented as mean ± SEM. *$p<0.05$, 2-tailed student's *t* test. Source data are provided as a Source Data file.

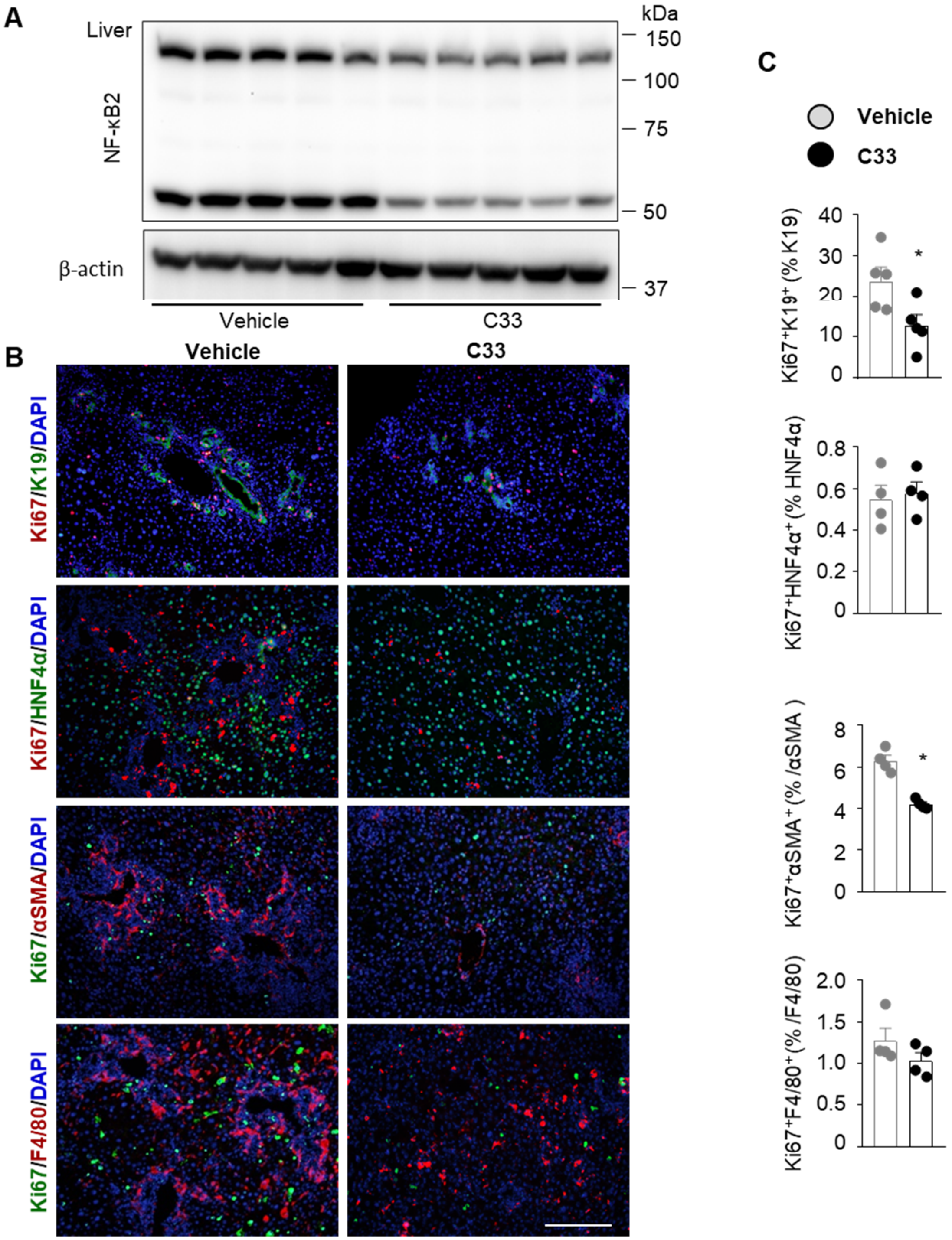


**Supplementary Figure 11. C33 treatment suppresses cholangiocyte and HSC proliferation in DDC-fed mice.** C57BL/6J male mice were fed a DDC diet and simultaneously treated with C33 (10 mg/kg, twice a week, i.p.) or vehicle for 3 weeks. **(A)** Liver extracts were immunoblotted with antibodies to NF-κB2 or β-actin. **(B-C)** Liver sections were stained with the indicated antibodies. Individual subpopulations were counted. $Ki67^{+}K19^{+}$: n=5 mice per group; the others: n=4 mice per group. Scale bar: 200 μm. Data are presented as mean ± SEM. *p<0.05, 2-tailed student's *t* test. Source data are provided as a Source Data file.

| ANTIBODY | SOURCE | Cat# | IHC | Blot |
|---|---|---|---|---|
| BrdU | Sigma | B2531 | 1000 | |
| Caspase-3 | Cell Signaling Technology | 9661 | 1000 | |
| CD11b | Biolegend | 101206 | 1000 | |
| CD4 | BD Biosciences | 553650 | 1000 | |
| CD8 | BD Biosciences | 552877 | 1000 | |
| F4/80 | eBioscience | 14-4801-82 | 1000 | |
| Gr-1 | Biolegend | 108408 | 1000 | |
| K19 | DSHB (University of Iowa) | Troma-III | 100 | |
| Ki67 | Vector lab | VP-RM04 | 1000 | |
| Ki67 | Invitrogen | 14-5698-92 | 1000 | |
| Myeloperoxidase | Thermo Fisher | RB-373-A0 | 1000 | |
| NF-κB2 | Cell Signaling Technology | 4882 | | 1000 |
| NIK | Abcam | ab191591 | 1000 | 1000 |
| NIK | Abcam | ab203568 | 300 | 5000 |
| p85 | home made | N/A | | 5000 |
| αSMA | Sigma | A5228 | 5000 | 5000 |
| TRAF2 | Cell Signaling Technology | 4712 | | 1000 |
| TRAF3 | Cell Signaling Technology | 4729 | | 1000 |
| cIAP1 | Cell Signaling Technology | 4952 | | 1000 |
| cIAP1 | ABclonal | A0866 | | 1000 |
| cIAP2 | Santa Cruz Biotechnology | sc-7944 | | 500 |
| cIAP2 | ABclonal | A0833 | | 1000 |
| β-actin | ABclonal | ac026 | | 20000 |
| HNF4α | Santa Cruz Biotechnology | sc-8987 | 1000 | |
| Akt | Cell Signaling Technology | 4691 | | 5000 |
| p-Akt | Cell Signaling Technology | 4060 | | 5000 |
| IKKα | Cell Signaling Technology | 2682 | 500 | |

**Supplementary Table 1. Antibody list.**

| Genes | Forward | Reverse |
|---|---|---|
| *18S* | CGCTTCCTTACCTGGTTGAT | GAGCGACCAAAGGAACCATA |
| *36B4* | AAGCGCGTCCTGGCATTGTCT | CCGCAGGGGCAGCAGTGGT |
| *Ccl2* | ACTGAAGCCAGCTCTCTCTTCCTC | TTCCTTCTTGGGGTCAGCACAGAC |
| *Ccl5* | CCACTTCTTCTCTGGGTTGG | GTGCCCACGTCAAGGAGTAT |
| *Cftr* | CTGGACCACACCAATTTTGAGG | GCGTGGATAAGCTGGGGAT |
| *Col1a1* | TCACCTACAGCACCCTTGTG | GGTGGAGGGAGTTTACACGA |
| *Ctgf* | TGCAGACTGGAGAAGCAGAG | GGCTTGGCGATTTTAGGTGT |
| *Hnf1*β | AGGGAGGTGGTCGATGTCA | TCTGGACTGTCTGGTTGAACT |
| *Il10* | CTGGACAACATACTGCTAACCG | GGGCATCACTTCTACCAGGTAA |
| *Il1*β | GCCTTGGGCCTCAAAGGAAAGAATC | GGAAGACACAGATTCCATGGTGAAG |
| *Il4* | CATGGGAAAACTCCATGCTT | TGGACTCATTCATGGTGCAG |
| *Il6* | AGCCAGAGTCCTTCAGA | GGTCCTTAGCCACTCCT |
| *iNos* | CAGGGCCACCTCTACATTTG-3 | TGCCCCATAGGAAAAGACTG-3 |
| *K19* | GGAAATTACTGCCCTGAGGAG | CTGGATCTGCTCAGAGTGGAC |
| *Mmp9* | CGTCGTGATCCCCACTTACT | AACACACAGGGTTTGCCTTC |
| *NIK (genotyping)* | CGAGGTCCACAGAATGAAGGAC | CAAGTCAGGGTCTCACAGCATAG |
| *NIK (qPCR)* | TCTCTGGAGGAACAGGAACAA | GCCATTGAGAGACTGGATCTG |
| *Sh2b2* | CAAATCAACAAGATGCACCG | CTTGGCCTTGCCCTGGAAGTTGAA |
| *Tgf*-β*1* | TTGCTTCAGCTCCACAGAGA | TGGTTGTAGAGGGCAAGGAC |
| Timp1 | GCTAAATTCATGGGTTCCCCAG | GAGAAAGCTCTTTGCTGAGCAG |
| *Tnf*α | CATCTTCTCAAAATTCGAGTGACAA | TGGGAGTAGACAAGGTACAACCC |
| *Vimentin* | GACCTCACTGCTGCCCTGCG | GACTCCTGCTTGGCCTGGCG |
| αSMA | GTTCAGTGGTGCCTCTGTCA | ACTGGGACGACATGGAAAAG |

**Supplementary Table 2. Primer list.**
