# Supplementary material for: Biliary NIK promotes ductular reaction and liver injury and fibrosis in mice
Source: Nat Commun. 2022 Aug 30;13:5111. doi: 10.1038/s41467-022-32575-8 (PMC9427946; doi:10.1038/s41467-022-32575-8)
Supplement: Supplementary file 3 — Reporting Summary [file 41467_2022_32575_MOESM3_ESM.pdf]

nature portfolio

Corresponding author(s): Liangyou Rui

Last updated by author(s): 5/18/2022



# Reporting Summary

Nature Portfolio wishes to improve the reproducibility of the work that we publish. This form provides structure for consistency and transparency in reporting. For further information on Nature Portfolio policies, see our Editorial Policies and the Editorial Policy Checklist.

## Statistics

For all statistical analyses, confirm that the following items are present in the figure legend, table legend, main text, or Methods section.

| n/a | Confirmed | |
|---|---|---|
| [ ] | [x] | The exact sample size ($n$) for each experimental group/condition, given as a discrete number and unit of measurement |
| [ ] | [x] | A statement on whether measurements were taken from distinct samples or whether the same sample was measured repeatedly |
| [ ] | [x] | The statistical test(s) used AND whether they are one- or two-sided<br>*Only common tests should be described solely by name; describe more complex techniques in the Methods section.* |
| [x] | [ ] | A description of all covariates tested |
| [x] | [ ] | A description of any assumptions or corrections, such as tests of normality and adjustment for multiple comparisons |
| [ ] | [x] | A full description of the statistical parameters including central tendency (e.g. means) or other basic estimates (e.g. regression coefficient) AND variation (e.g. standard deviation) or associated estimates of uncertainty (e.g. confidence intervals) |
| [ ] | [x] | For null hypothesis testing, the test statistic (e.g. $F$, $t$, $r$) with confidence intervals, effect sizes, degrees of freedom and $P$ value noted<br>*Give P values as exact values whenever suitable.* |
| [x] | [ ] | For Bayesian analysis, information on the choice of priors and Markov chain Monte Carlo settings |
| [x] | [ ] | For hierarchical and complex designs, identification of the appropriate level for tests and full reporting of outcomes |
| [x] | [ ] | Estimates of effect sizes (e.g. Cohen's $d$, Pearson's $r$), indicating how they were calculated |

*Our web collection on statistics for biologists contains articles on many of the points above.*

## Software and code

Policy information about availability of computer code

| | |
|---|---|
| Data collection | No software and code used for data collection. |
| Data analysis | GraphPad Prism 8 , Microsoft excel 2016 |

For manuscripts utilizing custom algorithms or software that are central to the research but not yet described in published literature, software must be made available to editors and reviewers. We strongly encourage code deposition in a community repository (e.g. GitHub). See the Nature Portfolio guidelines for submitting code & software for further information.

## Data

Policy information about availability of data

All manuscripts must include a data availability statement. This statement should provide the following information, where applicable:
- Accession codes, unique identifiers, or web links for publicly available datasets
- A description of any restrictions on data availability
- For clinical datasets or third party data, please ensure that the statement adheres to our policy

Source data are provided within this paper as a Source Data file.

# Field-specific reporting

# Life sciences study design

All studies must disclose on these points even when the disclosure is negative.

| | |
|---|---|
| Sample size | Sample size was calculated using N-query 6.0 with 80% power and 0.05 one-sided type I error to detect ~20% difference between mutant and control groups. Pilot studies were conducted to estimate sample size and calculate power of study. |
| Data exclusions | No data were excluded. |
| Replication | Authors confirm that data replication was successful. Experiments were conducted with calculated sample size for two or three times to ensure reproducibility. These independent experiments were combined when possible. Authors always had more than 3 samples for histology or immunohistochemistry to replicate results. |
| Randomization | Authors randomly allocated animals to experimental and control groups to make the experimental groups as similar as possible in all respects. In particular, the authors measured mouse body weight before the beginning of the experiment to make sure there is no weight difference between control and experimental groups. Other samples are also randomly allocated into appropriate experimental groups. |
| Blinding | Investigators were blinded to analysis whenever it is available. Immunohistochemistry images were quantified by non-study participants to reduce a possible bias. Blinding was used for animal data collection and analyses. |

# Reporting for specific materials, systems and methods

We require information from authors about some types of materials, experimental systems and methods used in many studies. Here, indicate whether each material, system or method listed is relevant to your study. If you are not sure if a list item applies to your research, read the appropriate section before selecting a response.

## Materials & experimental systems

| n/a | Involved in the study | |
|---|---|---|
| ☐ | ☒ | Antibodies |
| ☐ | ☒ | Eukaryotic cell lines |
| ☒ | ☐ | Palaeontology and archaeology |
| ☐ | ☒ | Animals and other organisms |
| ☐ | ☒ | Human research participants |
| ☒ | ☐ | Clinical data |
| ☒ | ☐ | Dual use research of concern |

## Methods

| n/a | Involved in the study | |
|---|---|---|
| ☒ | ☐ | ChIP-seq |
| ☒ | ☐ | Flow cytometry |
| ☒ | ☐ | MRI-based neuroimaging |

## Antibodies

| | |
|---|---|
| Antibodies used | BrdU (Sigma, B2531), Caspase3 (CST, #9661), CD11b (Biolegend, #101206), CD4 (BD Biosciences, #553650), CD8 (BD Biosciences, #552877), F4/80 (eBiosciences, #14-4801-82), Gr-1 (Biolegend, #108408), K-19 (DSHB, #TromaIII), Ki67 (Vector lab, #VP-RM04), Ki67 (Invitrogen, #14-5698-92), Myeloperoxidase (Thermo Fisher, #RB-373-A0), NF-κB2 (CST, #4882), NIK (Abcam, #ab191591), NIK (Abcam, #ab203568), p85 (generated by lab), αSMA (Sigma, A5228), TRAF2 (CST, #4712), TRAF3 (CST, #4729), cIAP1 (CST, #4952), cIAP1 (Abclonal, #A0866), cIAP2 (Santa Cruz, sc-7944), cIAP2 (Abclonal, #A0833), β-actin (Abclonal, #AC206), HNF4α (Santa Cruz, sc-8987), Akt (CST, #4691), pAKT (Ser473) (CST #4060), IKKα (CST, #2682). |
| Validation | Manufacturer's websites state all the antibodies are verified in terms of specificity, sensitivity, and reproducibility by following methods: Analysis of a large panel of cell lines with known target expression levels, Treatment of cells with appropriate kinase-specific activators and/or inhibitors, Phosphatase treatment, Correct subcellular localization or treatment-induced translocation, Comparison of results with antibody and isotype control to ensure acceptable signal-to-background ratio, Target-specific signal verified in transfected cells or knockout cells. Blocking with antigen peptide to confirm elimination of specific signal, Side-by-side comparison of a new lot with previous lots to ensure lot-to-lot consistency. |

## Eukaryotic cell lines

Policy information about cell lines

| | |
|---|---|
| Cell line source(s) | ATCC |
| Authentication | None of cell lines was authenticated. |
| Mycoplasma contamination | Negative |
| Commonly misidentified lines (See ICLAC register) | none |

## Animals and other organisms

Policy information about studies involving animals; ARRIVE guidelines recommended for reporting animal research

| | |
|---|---|
| Laboratory animals | Authors used age-matched littermates in C57BL/6J background to improve precision and reduce variability and bias. 8-week aged NIKf/f and NIKf/f;K19-CreERT males and females are used in this study. 8-week aged IKKαf/f and IKKαf/f;K19-CreERT males are used in this study. Males and females were characterized separately to determine the potential influence of sex on data interpretations. |
| Wild animals | This study does not involve wild animals. |
| Field-collected samples | This study does not involve field-collected samples. |
| Ethics oversight | Animal experiments were conducted following the protocols approved by the University of Michigan Institutional Animal Care and Use Committee (IACUC). |

Note that full information on the approval of the study protocol must also be provided in the manuscript.

## Human research participants

Policy information about studies involving human research participants

| | |
|---|---|
| Population characteristics | Both males and females at the age of over 30 years. |
| Recruitment | De-identified human liver specimens; patients provided informed consent; the double-blunted methods to avoid biases. |
| Ethics oversight | The study was approved by the Indiana-Purdue University Indianapolis and University of Michigan IRB committees. |

Note that full information on the approval of the study protocol must also be provided in the manuscript.
